# A novel *Rickettsia* subspecies closely related to *Rickettsia felis* in *Aedes albopictus* from Qingdao City, Eastern China

**DOI:** 10.3389/fcimb.2026.1814787

**Published:** 2026-06-02

**Authors:** Xin-Yi Zhang, Li-Zhu Fang, Bing-Hui Li, Rui Sun, Song Liu, Si-Tong Liu, Zhao-Guo Wang

**Affiliations:** 1 Department of Epidemiology and Health Statistics, The College of Public Health of Qingdao University, Qingdao, Shandong, China; 2 Qingdao Municipal Center for Disease Control and Prevention, Qingdao, Shandong, China

**Keywords:** Aedes albopictus, Candidatus Rickettsia felis subsp. laoshanensis, Eastern China, mosquitoes, Rickettsia felis

## Abstract

Mosquitoes are generally regarded as the main vectors for many zoonotic diseases, capable of transmitting various diseases and causing significant public health burdens. In this study, a total of 232 *Aedes albopictus* mosquitoes were captured from Qingdao City, Shandong Province in the eastern part of China, and it was believed that a new subspecies of *Rickettsia felis* had been discovered. Genetic analysis indicated that the *htrA* gene has highest 99.29% identity to *Rickettsia felis*, an emerging human pathogen attracting global attention, predominantly sustained by fleas, mosquitoes and booklice. The *16S* sequence of the strain had 100% of nucleotide similarity with *Rickettsia* sp. OnF11 gene for *16S rRNA*. The *gltA* sequence had 98.77% similarity to both Candidatus *Rickettsia senegalensis* isolate and Candidatus *Rickettsia senegalensis* strain PU01-02. The *groEL* sequence had 99.42% similarity to Candidatus *Rickettsia yingkouensis* clone YK50. The *ompB* sequence had 99.42% similarity to *Rickettsia hoogstraalii.* In the phylogenetic tree based on concatenated nucleotide sequences of *16S*, *gltA*, *groEL*, *htrA* and *ompB* genes, these strains were closely related to *R. felis*. Herein, it was named ‘Candidatus *Rickettsia felis* subsp. *laoshanensis*’. Its ability to induce pathogenesis in both human and animal hosts is yet to be comprehensively determined.

## 1 Introduction

More than 80% of the global population is at risk of contracting diseases transmitted by vectors, among which mosquito-borne diseases represent the largest factor in the burden of human vector-borne diseases (Franklinos et al., 2019; Ma et al., 2022; Abbasi, 2025; Hajissa et al., 2025). Mosquitoes belong to the family *Culicidae* within the order *Diptera* and exhibit hematophagous feeding habits. They are recognized as one of the most medically significant arthropods globally (Kadamov et al., 2012; Kirik et al., 2022; Petersen et al., 2024). There are over 3,500 described species of mosquitoes worldwide, mainly belonging to genera such as *Anopheles*, *Culex*, and *Aedes* (Wheelwright et al., 2021). *Anopheles* species like *A. sinensis* and *A. minutus* are the main vectors of malaria, *Culex* species like *C. pipiens molestus* and *C. pretorius* are important transmitters of lymphatic filariasis, and *Aedes* species like *A. aegypti* and *A. albopictus* are the main vectors of mosquito-borne viral diseases such as dengue fever, chikungunya fever, and Zika virus (Hall and Tamïr, 2022). In China, the complex ecological and topographical environment characterized by diverse host animals and land cover, combined with the rapid urbanization process and the increased human mobility and other changes in the behavior of large populations, have presented both opportunities and challenges for the study of the distribution and ecology of mosquitoes and mosquito-borne viruses (Liu et al., 2020).

*Rickettsia* spp. are obligate intracellular, Gram-negative bacteria that are primarily maintained in arthropod vectors (such as ticks, mites, fleas, and lice) and can be transmitted to humans, causing a group of diseases collectively known as rickettsioses (Qin et al., 2019; Zhang et al., 2023). In recent years, mosquitoes have also been reported to be the vectors for various *Rickettsiae* (Rymaszewska and Piotrowski, 2024; Xu et al., 2025), such as *Rickettsia felis* and *Rickettsia bellii* (Zhang et al., 2019b). In order to understand the current situation of *Rickettsia* in mosquitoes in China, we collected mosquitoes from Qingdao City, Shandong Province, and conducted *Rickettsia* tests on them.

## 2 Materials and methods

### 2.1 Mosquito samples

**Figure 1:**
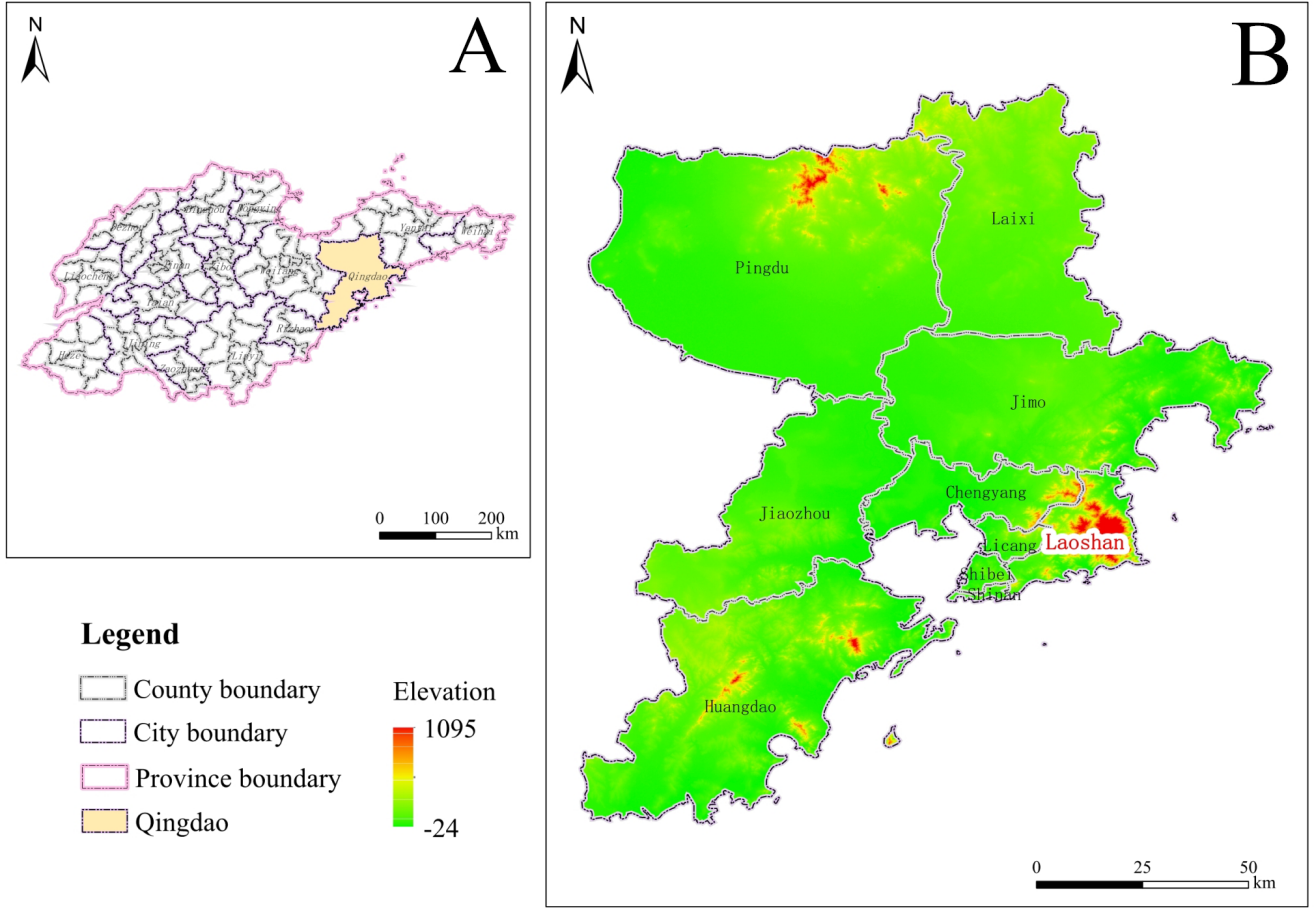
Geographic location and elevation distribution of the study area (Qingdao, Shandong Province, China). **(A)** The inset map illustrates the location of Qingdao within Shandong Province. **(B)** The elevation map presents the administrative divisions and topographic features of Qingdao, with elevation values ranging from -24 m to 1095 m as shown in the legend.

This study was conducted in Qingdao City, Eastern China (35°35′–37°09′ N, 119°30′–121°00′ E). Qingdao is a coastal city with a mixed urban-peri-urban-rural landscape, where the temperate monsoon climate favors mosquito breeding (**Figure 1**). Sporadic rickettsiosis cases have been documented locally, and *Aedes albopictus* is the dominant mosquito species. In August 2025, mosquitoes were collected in the districts of Chengyang, Shibei, Shinan, Pingdu and Laoshan of Qingdao using the double-layered tent trapping method and the human-baiting method. Specimens were preliminarily identified morphologically following standard taxonomic keys (Rueda, 2004), and species identity was further confirmed by amplification and sequencing of the cytochrome oxidase subunit I (COI) gene.

Mosquito individuals collected from the same sampling site were grouped and processed in pools for subsequent detection. After rinsing with PBS, each mosquito sample was placed into a homogenization tube (Roche Diagnostics GmbH, Hilden, Germany), followed by the addition of 1000 μL high-glucose DMEM medium (Gibco, USA) and metal beads (Hilden, Germany). Samples were homogenized in a tissue grinder (Hilden, Germany) at 7000 rpm for 30 s. Subsequently, 200 μL of the resultant homogenate was mixed with 250 μL of lysis buffer (Hilden, Germany). Nucleic acid extraction was then performed using an automated nucleic acid extractor (Hilden, Germany), yielding 200 μL of purified nucleic acid per sample. All nucleic acid samples were stored at -80 °C refrigerator before use.

### 2.2 PCR amplification of Mosquitoborne pathogens in mosquitoes

Firstly, a semi-nested PCR was performed on a conserved region of the *16S* gene (900bp) to detect the *Rickettsia*. To further analyze the pathogen, the sequences of a longer *16S* (1200bp), *gltA* (citrate synthase), *groEL* (60kDa chaperonin) (Lu et al., 2022), *htrA* (17kDa antigen) and *ompB* (outer membrane protein B) (Borsoi et al., 2019) genes were successfully obtained (The primer sequences and annealing temperatures can be found in **Supplementary Table 1**). To minimize the risk of PCR false positives, DNA extraction, reaction setup preparation, PCR amplification, and amplicon analysis were performed in physically separated rooms. First-round PCR was performed in a total volume of 20 μL, containing 10 μL of 2× Taq Master Mix (Dye Plus) (Nanjing Vazyme Biotech Co., Ltd., China), 2 μL forward primer, 2 μL reverse primer, 2 μL template DNA, and 4 μL nuclease-free water. The second-round PCR adopted an identical reaction system, with 2 μL of the first-round PCR product substituted for the original template DNA. Both PCR rounds shared the same thermal profile: initial denaturation at 95 °C for 3 min; 35 cycles of 95 °C for 15 s, 50 °C annealing for 15 s, and 72 °C elongation for 1 min; followed by a final extension at 72 °C for 7 min. Nuclease-free water was used as the negative control in all PCR assays. All PCR products were separated via 1% agarose gel electrophoresis, and amplicons with suspected positive results were subjected to Sanger sequencing (Tsingke Biotechnology Co., Ltd., Beijing, China).

### 2.3 Phylogenetic analysis

The sequences were examined and analyzed using the Chromas and BLAST programs (http://blast.ncbi.nlm.nih.gov/Blast.cgi). Sequence alignments of five genes (*16S rRNA*, *gltA*, *groEL*, *htrA*, and *ompB*) were separately performed using MEGA 7 software. After manual trimming of low-variability regions, the qualified individual gene alignments were concatenated head-to-tail to generate a combined multi-gene tandem dataset. The dataset was subsequently used for phylogenetic reconstruction, and the phylogenetic tree was constructed employing the maximum likelihood method. The bootstrap values, which was calculated with 1000 repetitions, were only displayed exceeding 60%. All the obtained sequences have been submitted to the GenBank database (Accession numbers: PX704800, PX797672, PX698602-PX698604).

## 3 Results

### 3.1 Mosquito species

A total of 232 mosquito individuals were collected during the investigation. All specimens were identified as *Aedes albopictus* via morphological observation and COI gene sequencing verification. According to the sampling location, all specimens were grouped into 26 pools in total. The pool positive rate was 3.85% for each gene, and the overall minimum infection rate (MIR) of mosquitoes was 0.43%. Summary data of mosquito samples and pathogen screening are provided in **Table 1**.

**Table 1:** Summary of mosquito samples and *Rickettsia* detection results.

Index	Detailed information	Value	Positive rate (%)
Total tested mosquitoes	Individuals/Pools	232/26	–
*Rickettsia*-positive samples	Positive pools/Mosquitoes in positive pool	1/8	–
Overall infection rate	Minimum infection rate	–	0.43
Gene-specific positive rate*	*16S rRNA*, *gltA*, *groEL*, *htrA*, *ompB*	1/26	3.85

*All five target genes were detected positive in the same single pool.

### 3.2 Phylogenetic analysis of *Rickettsia*

All the mosquito samples were initially subjected to a semi-nested PCR based on the *16S* gene for testing. The result showed that one of the pools tested positive, and there were 8 mosquitoes in this positive pool. To further characterize, the sequences of a longer *16S*, *gltA*, *groEL*, *htrA* and *ompB* genes were successfully obtained. All the obtained sequences have been submitted to the GenBank database (Accession numbers: PX704800, PX797672, PX698602-PX698604).

Genetic analysis indicated that the *htrA* gene has highest 99.29% identity to *Rickettsia felis*, an emerging human pathogen attracting global attention, predominantly sustained by fleas, mosquitoes and booklice. The *16S rRNA* gene sequence of the strain exhibited 100% nucleotide identity with *Rickettsia* sp. OnF11, while the *gltA*, *groEL*, and *ompB* gene sequences showed similarities of 98.77% (with both Candidatus *Rickettsia senegalensis* isolate and strain PU01-02), 99.42% (with Candidatus *Rickettsia yingkouensis* clone YK50), and 99.42% (with *Rickettsia hoogstraalii*), respectively.

**Figure 2:**
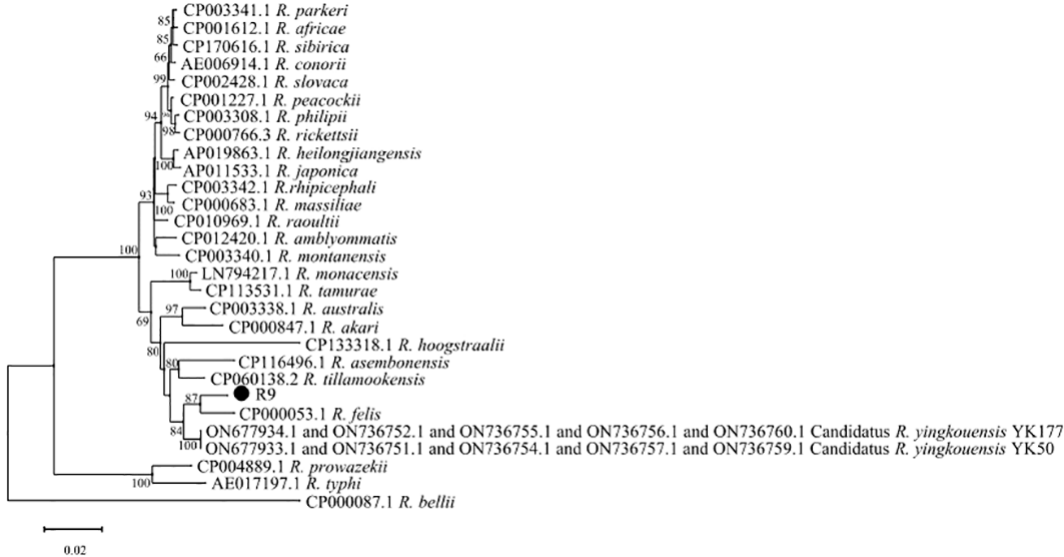
Phylogenetic tree of the concatenation of *Rickettsia 16S*, *gltA*, *groEL*, *htrA* and *ompB* gene. The tree was generated using the Maximum Likelihood method, and 1000 replicates for bootstrap testing in MEGA 7.0 software. Only bootstrap values > 60% were shown. *Rickettsia* sequences obtained in this study are shown with dots. The scale bar indicates nucleotide substitutions per site. The *Rickettsia* species’ name and complete genome GenBank accession numbers of reference sequences are shown in each line.

The concatenated phylogenetic tree, constructed based on the partial sequences of the *16S* (1200 bp), *gltA* (1100 bp), *groEL* (800 bp), *htrA* (400 bp) and *ompB* (400 bp) genes, demonstrated that the strain forms a distinct branch, which was closely related to the known human pathogen *Rickettsia felis* (**Figure 2**). And based on the sequence-based criteria established in the previous article (Fournier et al., 2003) for the identification of novel *Rickettsia* subspecies, it was proposed that these strains represented a new *Rickettsia felis* subspecies. Since this strain was identified solely by molecular detection and has not been isolated in pure culture, it was tentatively designated as ‘Candidatus *Rickettsia felis* subsp. *laoshanensis*’, based on the geographical location of its initial discovery. The phylogenetic tree of a single system consisting of five genes had also been completed (**Supplementary Figures 1**-**5**).

## 4 Discussion

232 mosquitoes were collected from 5 districts in Qingdao City, Shandong Province, China and all the mosquitoes were *Aedes albopictus*. They were divided into 26 pools based on the capture location and their own characteristics. These 26 pools were subjected to PCR testing for the *16S* gene (900 bp) of *Rickettsia*, the results showed that one of the pools was positive. The positive pool was subjected to a longer *16S* (1200bp), *gltA*, *groEL*, *htrA* and *ompB* tests for *Rickettsia*. It was believed that a new subspecies of *Rickettsia felis* had been discovered, and it was named “Candidatus *Rickettsia felis* subsp. *laoshanensis*” after the location where it had been first discovered.

*Rickettsia* is an intracellular gram-negative alpha-proteobacterium that can cause zoonotic cat flea typhus fever in humans and animals (Qin et al., 2019), also known as human flea-transmitted spotted fever. In 1990, Adams et al. (1990) observed it in the midgut epithelial cells of cat fleas (*Ctenocephalides felis*) using an electron microscope, and in 1995, based on its phenotypic characteristics and significant genetic differences from other known *Rickettsia* species, it was proposed to be named *Rickettsia felis* (Higgins et al., 1996). The foreign research covers a wide range of areas, including Southeast Asia, Europe, Africa, and others. Suporn Foongladda et al. (2011) examined flea samples from cats and dogs in Bangkok, Thailand, and reported that 67.4% of the canine flea samples contained rickettsiae related to cat fleas. In a separate investigation conducted in Zambia (Moonga et al., 2019), Lavel Chinyama Moonga et al. screened local dogs, rodents, and cat fleas for *Rickettsia felis* infection, documenting positive rates of 4.7% in canine blood samples, 11.3% in rodent tissue samples, and 3.7% in cat flea samples. A study conducted in Saint Kitts, USA, was the first to detect *R.felis* in *Culex quinquefasciatus*, *Aedes aegypti*, and *Aedes taeniorhynchus* (Valentine et al., 2021). Furthermore, *Rickettsia felis* were also detected in American opossums, German hedgehogs (Williams et al., 1992), wild boars and cattle in Laos (Kernif et al., 2012), as well as in wild African apes (Editors, 2024), etc.Francisco J Márquez et al. (2002) were the first to detect cat flea *rickettsiae* in cat fleas in southwestern Spain, which is the first recorded discovery of this pathogen on the Eurasian continent. Researches on *Rickettsia felis* in China focus on the pathogen carrying status of vector hosts (such as cat fleas, ticks, lice, etc.), diagnosis and treatment of human infection cases, and analysis of epidemic characteristics (Zhang et al., 2019b; Zhang et al., 2019a). Jiangsu Province was identified as the first location where *Rickettsia felis* was discovered in China. This discovery was derived from the research conducted by Jilei Zhang et al., and simultaneously, the ability of *Rickettsia felis* to infect humans in China was first documented in this study (Zhang et al., 2014). In our study, a novel subspecies named ‘Candidatus *Rickettsia felis* subsp. *laoshanensis*’ closely related to *R. felis* was identified in *Aedes albopictus*, one of the dominant mosquito species in Qingdao city, Shandong province. In 2023, in Yingkou, Liaoning Province, a closely related new strain of *R. felis*, designated as ‘Candidatus *Rickettsia yingkouensis*’, was detected in both *Anopheles sinensis* and *Anopheles pullus* (Lu et al., 2023). Compared with Candidatus *Rickettsia yingkouensis*, a closer genetic relationship is exhibited by our sequence with *Rickettsia felis*.

Mosquitoes are the most important vector organisms for disease transmission worldwide. As a dominant mosquito species in Qingdao City, *Aedes albopictus* is a species of mosquito belonging to the *Culicidae* family and the *Aedes* genus. It is a highly aggressive diurnal mosquito (Laojun and Chaiphongpachara, 2025). Originating from Southeast Asia, this species has undergone extensive global invasion, colonizing all continents except Antarctica. Its distribution spans tropical regions of South America and Africa, as well as most temperate areas in North America and Europe, and it is universally acknowledged as a major invasive species with significant implications for public health worldwide (Bonizzoni et al., 2013; Goubert et al., 2016; Motoki et al., 2019). *Aedes albopictus* is the primary vector for a variety of human arboviruses, including dengue, chikungunya, Zika, yellow fever, etc., making its impact far greater than that of ordinary mosquitoes (Zhong et al., 2013; Enserink, 2015; Ngoagouni et al., 2015; Amraoui et al., 2016; Battaglia et al., 2016). The cat flea has long been recognized as the primary traditional vector of *Rickettsia felis*, whereas research investigating the potential role of *Aedes albopictus* in transmitting this pathogen remains relatively scarce. A pioneering study conducted in Libreville, Gabon, employed specialized detection methodologies to identify *R. felis* in local populations of *A. albopictus* (Socolovschi et al., 2012). This groundbreaking finding challenges the long-standing paradigm that the cat flea serves as the exclusive primary vector of *R. felis*, thereby implying that *A. albopictus* could function as a potential transmission vector for this pathogen. In doing so, this work provides novel insights and opens up new avenues for future research into the transmission ecology of *R. felis*. These results collectively lend support to the hypothesis that *Aedes albopictus* might contribute to the transmission of human pathogenic *Rickettsia*. While it remains unclear whether ‘Candidatus *Rickettsia felis* subsp. *laoshanensis’* serves solely as an endosymbiont of this mosquito species or also exhibits pathogenic potential in animals, the substantial population size and strong aggressive feeding behavior of *Aedes albopictus* could facilitate frequent contact between this *Rickettsia* strain and humans as well as other vertebrate hosts. Due to its high similarity in genes to *R. felis*, its pathogenicity to humans still requires further study.

Nevertheless, several limitations remain in this study. The sampling was limited to a single location with a relatively small mosquito sample size, which may restrict the generalizability of the results. In addition, identification and phylogenetic analysis relied only on partial fragments of the target genes, without further functional or pathogenicity evaluation. Even so, our data provide baseline information for local *Rickettsia* surveillance. Future work shall expand sampling across more sites and time points to further validate the prevalence of this *Rickettsia* subspecies.

## Data Availability

The datasets presented in this study can be found in online repositories. The names of the repository/repositories and accession number(s) can be found in the article/**Supplementary Material**.
