# Supplementary material for: A novel Rickettsia subspecies closely related to Rickettsia felis in Aedes albopictus from Qingdao City, Eastern China
Source: Front Cell Infect Microbiol. 2026 Jun 2;16:1814787. doi: 10.3389/fcimb.2026.1814787 (PMC13268991; doi:10.3389/fcimb.2026.1814787)
Supplement: Supplementary file 1 [file DataSheet1.docx]

Supplementary Material

# Supplementary Table 1

**Table 1.** Primers for the amplification of sequences of *Rickettsia 16S*, *gltA*, *groEL*, *htrA* and *ompB* genes from mosquitoes.

| **Organisms** | **Primary/Nested** | **Primers** | **annealing temperature** | **Primer sequences** | **Target gene** | **Amplicon size** | **Reference** |
| --- | --- | --- | --- | --- | --- | --- | --- |
| *Rickettsia* | Primary | Ric-F | 50℃ | YTACGGAATAACTTTTAGAAA | *16S* | 900 bp | Lu et al., 2022 |
|  | Nested | Ric-R1 | 50℃ | CATGATGACTTGACRTCGT |  |  |  |
|  |  | Ric-R2 | 50℃ | CATCTCACGACACGAGCTG |  |  |  |
|  | Primary | Ric-glt-F1 | 52℃ | ACTTAYGAYCCGGGCTTTAT | *gltA* | 1100 bp |  |
|  |  | Ric-glt-F2 | 52℃ | CTTTATGTCTACTGCTTCTTG |  |  |  |
|  | Nested | Ric-glt-R | 52℃ | AGCTGTCTAGGTCTGCTGATT |  |  |  |
|  | Primary | Ric-gro-F1 | 52℃ | CCATTACATGATAGAATTGCAA | *groEL* | 800 bp |  |
|  |  | Ric-gro-F2 | 52℃ | GAATTGCAATAAAGCCTATCG |  |  |  |
|  | Nested | Ric-gro-R | 52℃ | CCATCATTGCTTTTCTTCTATC |  |  |  |
|  | Primary | htrAex5 | 53℃ | GCTTTACAAAATTCTAAAAACCATATA | *htrA* | 400 bp | Borsoi et al.,  2019 |
|  |  | htrAex3 | 53℃ | TGTCTATCAATTCACAACTTGC |  |  |  |
|  | Nested | htrAin5 | 48℃ | GCTCTTGCAACTTCTATGT |  |  |  |
|  |  | htrAin5 | 48℃ | CATTGTTCGTCAGGTTGGC |  |  |  |
|  | Primary | ompBex5 | 54℃ | GTAACCGGAAGTAATCGTTTCGTAA | *ompB* | 400 bp |  |
|  |  | ompBex3 | 54℃ | CTTTATAACCAGCTAAACCAC |  |  |  |
|  | Nested | ompBin5 | 51℃ | GTTTAATACGTGCTGCTAACCA |  |  |  |
|  |  | ompBin3 | 51℃ | GGTTTGGCCCATATACCATAA |  |  |  |

*Degenerate primer: Y = C or T.

# Supplementary Figures

***Rickettsia 16S* gene**

DNA sequence analysis indicated that the positive sample has the highest homology with *Rickettsia* sp. OnF11 gene for *16S* rRNA (GenBank: LC558311.1) (100%). The *16S*-9 represented the amplified positive sequence of the *16S* gene. The phylogenetic analysis of the *16S* sequence showed that the positive sample is closely related to *R. tillamookensis.*


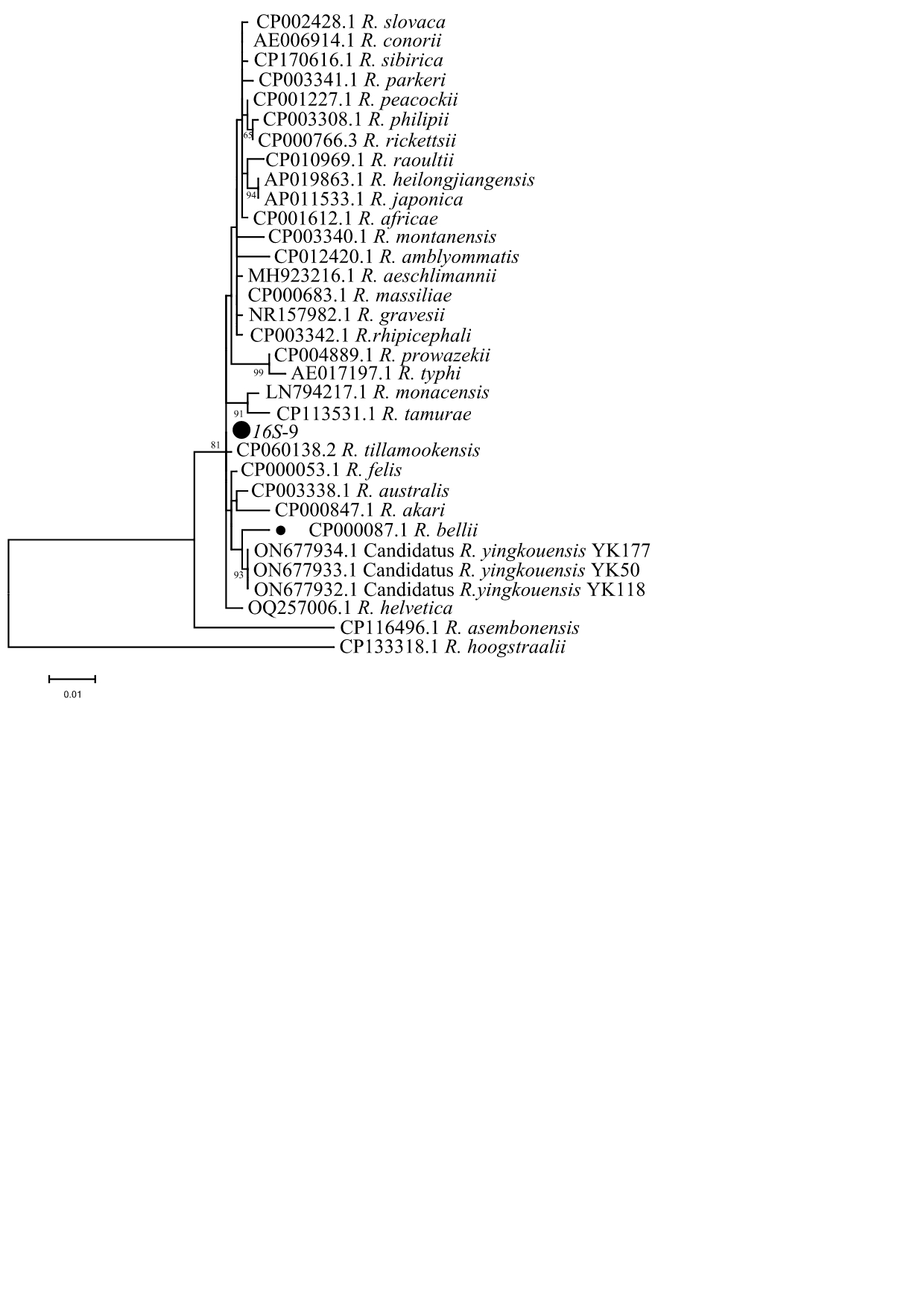


**Supplementary Figure 1.** Phylogenetic tree of the *Rickettsia 16S* gene. The tree was generated using the Maximum Likelihood method, and 1000 replicates for bootstrap testing in MEGA 7.0 software. Only bootstrap values > 60% were shown. *Rickettsia* sequences obtained in this study are shown with dots. The scale bar indicates nucleotide substitutions per site. The *Rickettsia* species' name and complete genome GenBank accession numbers of reference sequences are shown in each line.

***Rickettsia gltA* gene**

DNA sequence analysis indicated that the positive sample has the highest homology with Candidatus *Rickettsia* *senegalensis* isolate RAPCF15 citrate synthetase (*gltA*) gene (GenBank: KU499847.1) and Candidatus *Rickettsia* *senegalensis* strain PU01-02 citrate synthase (*gltA*) gene (GenBank: KF666472.1) (98.77%). The *gltA*-9 represented the amplified positive sequence of the *gltA* gene. The phylogenetic analysis of the *gltA* sequence indicated that the positive sample has a closest genetic relationship with *R. aeschlimannii.*


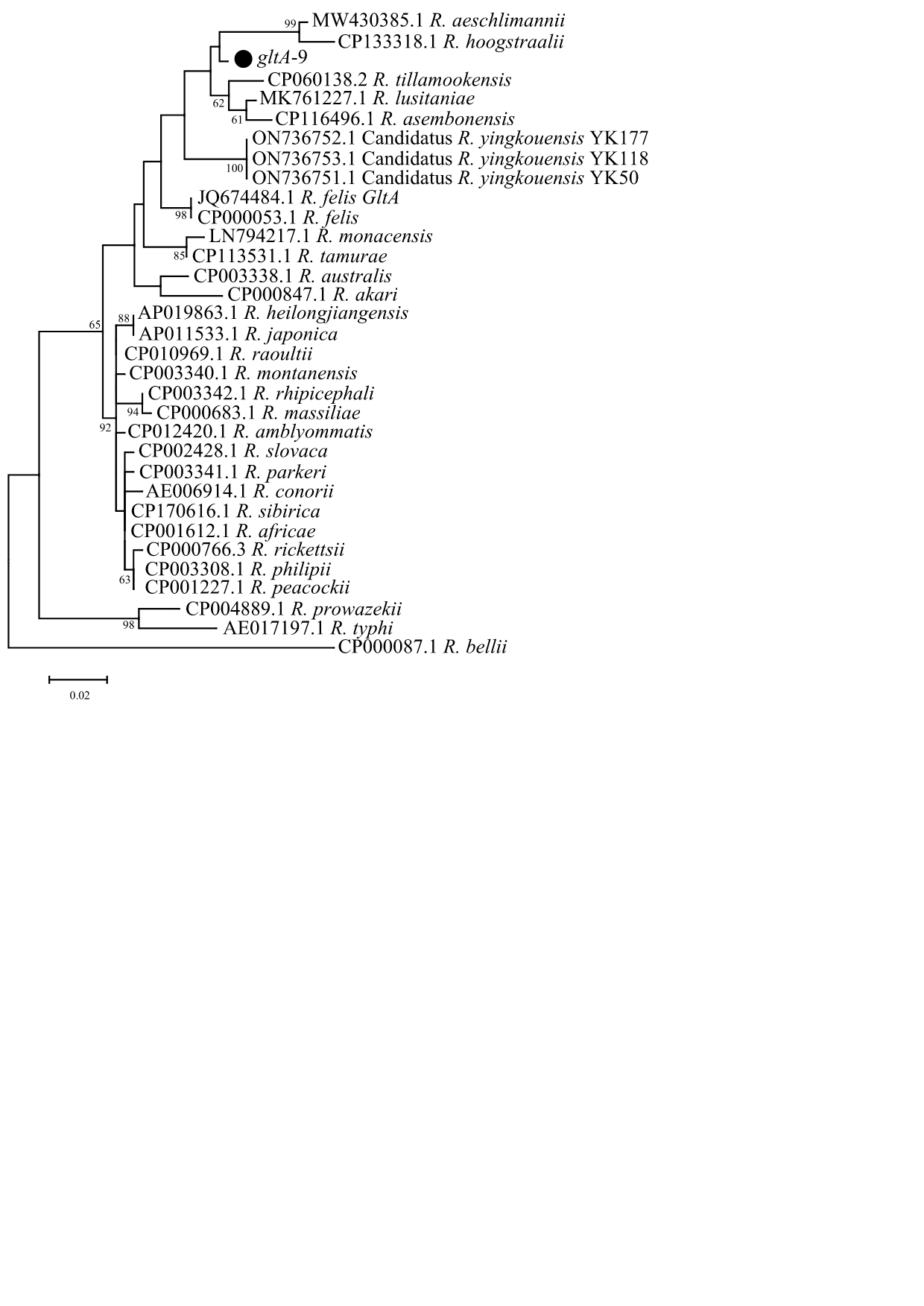


**Supplementary Figure 2.** Phylogenetic tree of the *Rickettsia gltA* gene. The tree was generated using the Maximum Likelihood method, and 1000 replicates for bootstrap testing in MEGA 7.0 software. Only bootstrap values > 60% were shown. *Rickettsia* sequences obtained in this study are shown with dots. The scale bar indicates nucleotide substitutions per site. The *Rickettsia* species' name and complete genome GenBank accession numbers of reference sequences are shown in each line.

***Rickettsia groEL gene***

DNA sequence analysis indicated that the positive sample has the highest homology with Candidatus *Rickettsia yingkouensis* clone YK50 *GroEL* (*groEL*) and *GroES* (*groES*) genes (GenBank: ON736754.1) (99.42%). The *groEL*-9 represented the amplified positive sequence of the *groEL* gene. The phylogenetic analysis of the *groEL* sequence indicated that the positive sample has a close genetic relationship with *R. felis*.


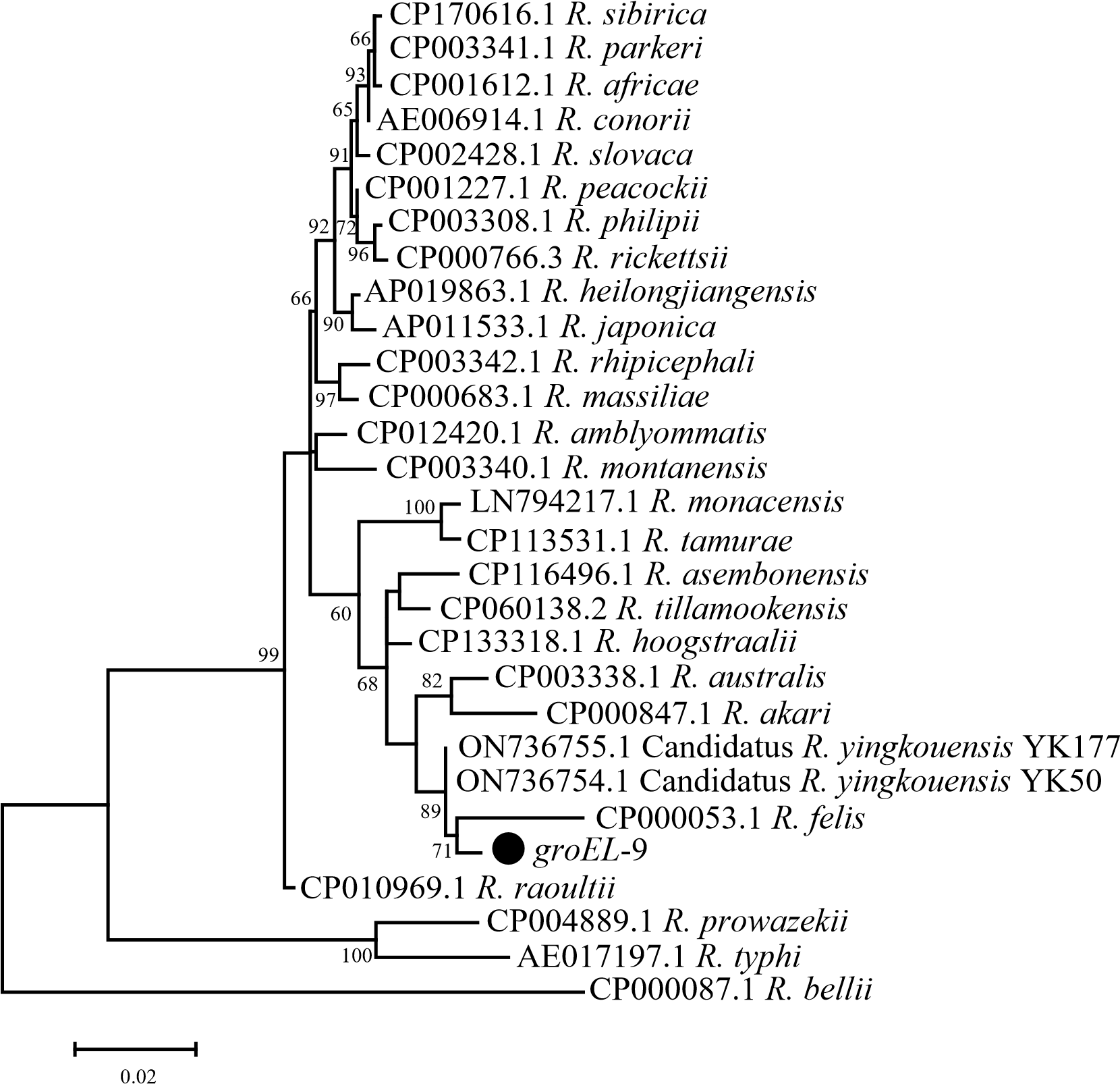


**Supplementary Figure 3.** Phylogenetic tree of the *Rickettsia groEL* gene. The tree was generated using the Maximum Likelihood method, and 1000 replicates for bootstrap testing in MEGA 7.0 software. Only bootstrap values > 60% were shown. *Rickettsia* sequences obtained in this study are shown with dots. The scale bar indicates nucleotide substitutions per site. The *Rickettsia* species' name and complete genome GenBank accession numbers of reference sequences are shown in each line.

***Rickettsia htrA gene***

DNA sequence analysis indicated that the positive sample has the highest homology with *R. felis* (GenBank: LC764839.1, ON209420.1, MK509750.1, MG818716.1) (99.29%). The *htrA*-9 represented the amplified positive sequence of the *htrA* gene. The phylogenetic analysis of the *htrA* sequence indicated that the positive sample has a close genetic relationship with *R. felis*.


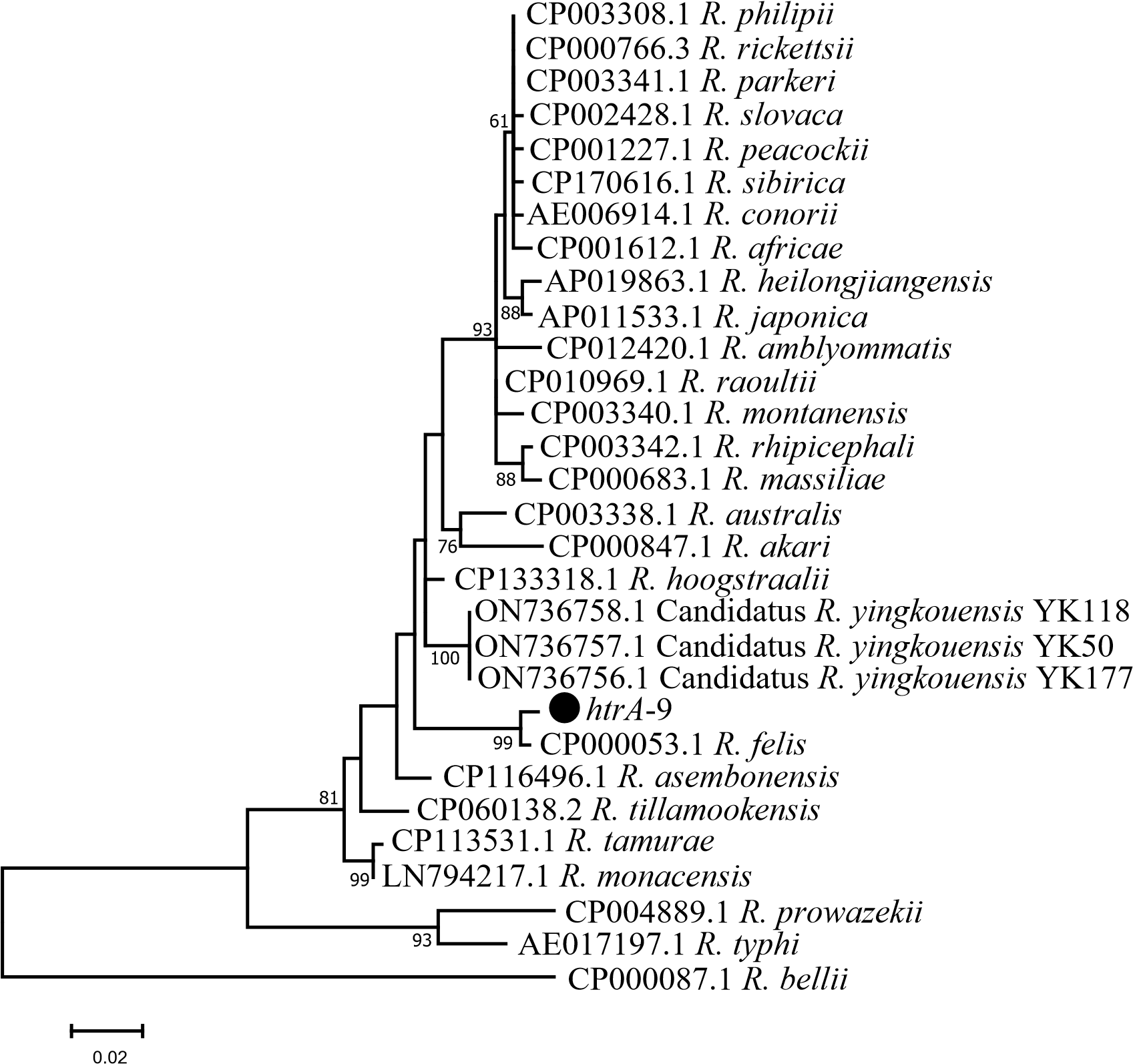


**Supplementary Figure 4.** Phylogenetic tree of the *Rickettsia htrA* gene. The tree was generated using the Maximum Likelihood method, and 1000 replicates for bootstrap testing in MEGA 7.0 software. Only bootstrap values > 60% were shown. *Rickettsia* sequences obtained in this study are shown with dots. The scale bar indicates nucleotide substitutions per site. The *Rickettsia* species' name and complete genome GenBank accession numbers of reference sequences are shown in each line.

***Rickettsia ompB gene***

DNA sequence analysis indicated that the positive sample has the highest homology with *R. hoogstraalii* (GenBank: PQ152000.1) (96.77%). The *ompB*-9 represented the amplified positive sequence of the *ompB* gene. The phylogenetic analysis of the *ompB* sequence indicated that the positive sample has a close genetic relationship with Candidatus *R. yingkouensis* YK118.


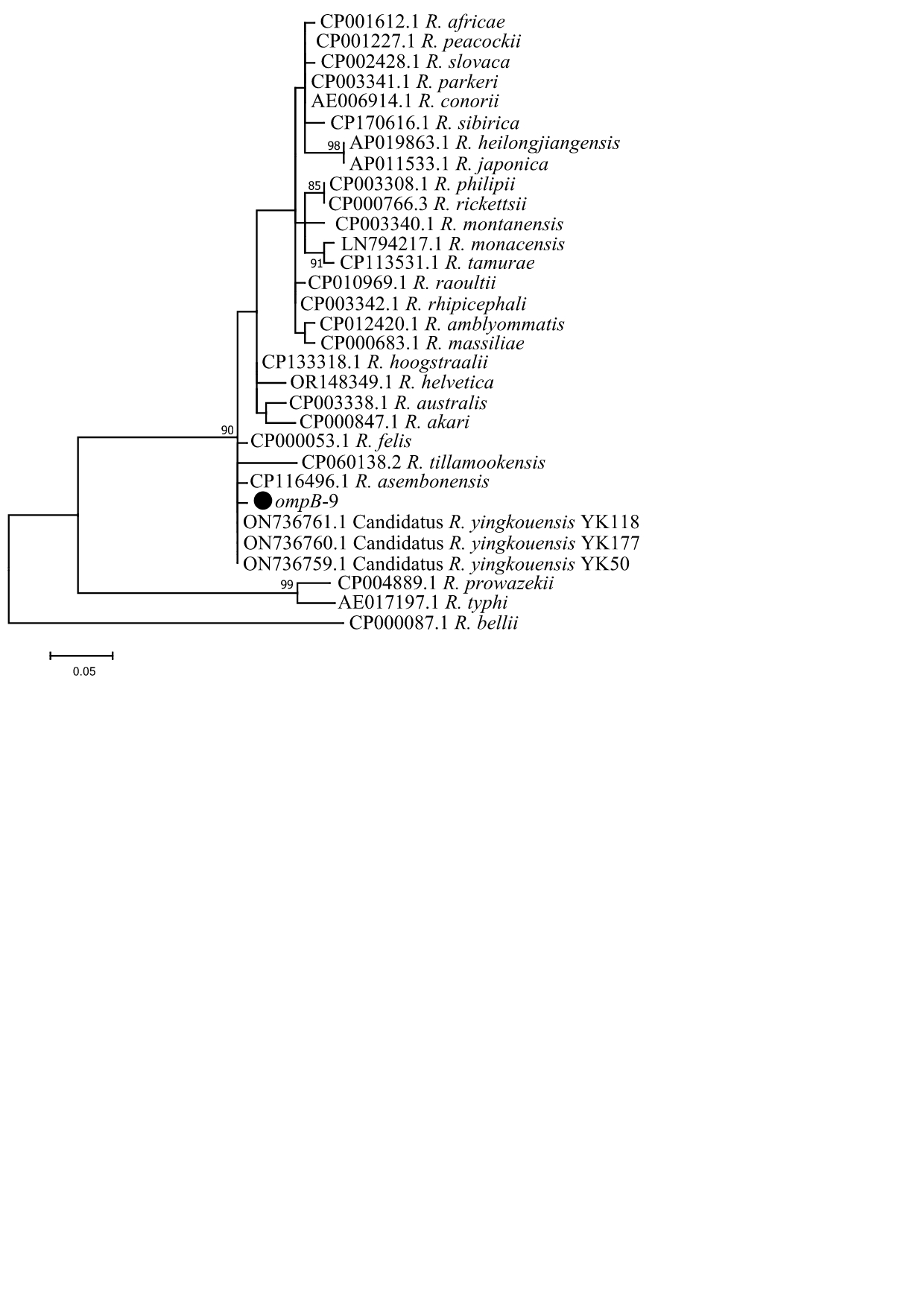


**Supplementary Figure 5.** Phylogenetic tree of the *Rickettsia ompB* gene. The tree was generated using the Maximum Likelihood method, and 1000 replicates for bootstrap testing in MEGA 7.0 software. Only bootstrap values > 60% were shown. *Rickettsia* sequences obtained in this study are shown with dots. The scale bar indicates nucleotide substitutions per site. The *Rickettsia* species' name and complete genome GenBank accession numbers of reference sequences are shown in each line.
